# Unexpected Dual Task Benefits on Cycling in Parkinson Disease and Healthy Adults: A Neuro-Behavioral Model

**DOI:** 10.1371/journal.pone.0125470

**Published:** 2015-05-13

**Authors:** Lori J. P. Altmann, Elizabeth Stegemöller, Audrey A. Hazamy, Jonathan P. Wilson, Michael S. Okun, Nikolaus R. McFarland, Aparna Wagle Shukla, Chris J. Hass

**Affiliations:** 1 Department of Speech, Language, and Hearing Sciences, University of Florida, Gainesville, Florida, United States of America; 2 Department of Applied Physiology and Kinesiology, University of Florida, Gainesville, Florida, United States of America; 3 Department of Neurology and Neurosurgery, University of Florida, Gainesville, Florida, United States of America; 4 Center for Movement Disorders and Neurorestoration, University of Florida, Gainesville, Florida, United States of America; 5 Department of Kinesiology, Iowa State University, Ames, Iowa, United States of America; 6 Department of Speech-Language Pathology, Midwestern University, Downers Grove, Illinois, United States of America; Anhui Medical University, CHINA

## Abstract

**Background:**

When performing two tasks at once, a dual task, performance on one or both tasks typically suffers. People with Parkinson’s disease (PD) usually experience larger dual task decrements on motor tasks than healthy older adults (HOA). Our objective was to investigate the decrements in cycling caused by performing cognitive tasks with a range of difficulty in people with PD and HOAs.

**Methods:**

Twenty-eight participants with Parkinson’s disease and 20 healthy older adults completed a baseline cycling task with no secondary tasks and then completed dual task cycling while performing 12 tasks from six cognitive domains representing a wide range of difficulty.

**Results:**

Cycling was faster during dual task conditions than at baseline, and was significantly faster for six tasks (all *p*<.02) across both groups. Cycling speed improved the most during the easiest cognitive tasks, and cognitive performance was largely unaffected. Cycling improvement was predicted by task difficulty (*p*<.001). People with Parkinson’s disease cycled slower (p<.03) and showed reduced dual task benefits (p<.01) than healthy older adults.

**Conclusions:**

Unexpectedly, participants’ motor performance improved during cognitive dual tasks, which cannot be explained in current models of dual task performance. To account for these findings, we propose a model integrating dual task and acute exercise approaches which posits that cognitive arousal during dual tasks increases resources to facilitate motor and cognitive performance, which is subsequently modulated by motor and cognitive task difficulty. This model can explain both the improvement observed on dual tasks in the current study and more typical dual task findings in other studies.

## Introduction

Everyday life often requires performing two or more tasks at once (i.e. dual tasking), such as walking while talking to a friend. Inability to efficiently or effectively perform concurrent tasks can impact quality of life and limit participation in community activities [1–3]. In the overwhelming majority of dual task research, performance on one or both tasks worsens in healthy participants when two tasks are performed simultaneously [4,5]. These changes in performance are called dual task effects (DTEs) [6–8]. Explanations for changes in performance during or immediately after exercise often refer to Kahneman’s model of attention [9,10]. In this model, which is based on psychological rather than neurological principles, both motor and cognitive tasks tap cognitive resources, such as attention [7,8,10], working memory [3] and executive function [8], all of which are inherently limited [8,11]. Therefore, when the total cognitive demands of the two tasks exceed available resources, performance on one or both tasks suffers. Three important implications follow from this hypothesis. First, the difficulty of the tasks employed should predict the magnitude of DTEs: pairing more difficult tasks should yield greater DTEs. Second, DTEs should be greater in populations with reduced resources due to neurologic disorders, such as people with Parkinson’s disease (PD). Third, persons who are already impaired in the motor task should show the greatest DTEs [12].

Recently, Al-Yahya et al.[6] performed a meta-analysis to quantify the magnitude of DTEs on gait caused by tasks from varying cognitive domains. Al-Yahya et al. categorized studies by type of cognitive task employed (in increasing order of difficulty): response time, discrimination and decision making (controlled processing), working memory, mental tracking (updating, executive function), and verbal fluency. Although they found that all cognitive tasks were associated with DTEs, the strongest, most consistent dual task effects were found during executive function and working memory tasks. However, to date no within-subjects study, in which the same participants complete multiple cognitive tasks in single and dual task conditions, has examined the magnitude of DTEs associated with these cognitive domains on gait or any other motor task. The current study, using cycling as the motor task, compares the magnitude of DTEs across twelve cognitive tasks chosen to reflect a similar continuum of difficulty as presented in Al-Yahya et al.

Previous research documents that DTEs on gait and balance in people with PD exceed those in healthy older adults (HOAs). This result is so common that O’Shea et al. call the presence of unusually high DTEs a characteristic feature of PD [13]. However, these studies employ a limited range of motor and cognitive tasks, focusing primarily on gait and balance paired with complex executive function tasks [7,14]. Importantly, walking and maintaining balance are impaired in PD even with no secondary task, due to degeneration of basal ganglia regions for motor control. This degeneration causes walking and balance control to lose automaticity and become more attention-demanding [3,14–17]. Thus, when additional demands are imposed by a simultaneous cognitive task, motor control becomes increasingly vulnerable. The current study extends this literature by contrasting performance in people with PD and HOAs using cycling, which is not impaired in PD [18,19], paired with a wide range of secondary cognitive tasks.

Evaluations of DTEs across tasks frequently compare across modalities [20], for example comparing the effects of a cognitive versus a motor task on gait, which renders a priori determination of task difficulty impossible. The aim of this study is to provide a direct comparison of the magnitude of DTEs on cycling in PD and HOAs, within-subjects, elicited by twelve tasks representing a broad range of difficulty within one modality, cognition. This will allow a direct test of the assumption that more difficult secondary tasks lead to greater DTEs, while identifying tasks that cause significant DTEs on cycling. Based on current literature, three hypotheses are tested in this study: 1) cycling will slow during dual task performance of the most difficult cognitive tasks relative to baseline (i.e., single task) cycling, 2) DTEs will be greater in PD than in healthy older adults, and 3) DTEs on cycling will be predicted by the hypothesized difficulty of the cognitive tasks.

## Materials and Methods

### Participants

Forty individuals with PD were recruited from the Center for Movement Disorders and Neurorestoration in Gainesville, Florida, and twenty healthy older adults (HOAs) were recruited from the surrounding community. Two individuals with PD were excluded for not completing both the single and dual task sessions. Five individuals from the PD group were unable to complete the most difficult cognitive task (Two-back) and so were excluded from the study, and an additional five individuals with PD were excluded due to missing cycling data in one or more tasks. Missing cycling data were due to marker occlusion caused by research staff acting as spotters being positioned near participants on the bicycle, which obstructed the view from the cameras. Spotters were not necessary with the HOA participants, but were for some PD participants. Thus, data from 28 individuals with PD and 20 HOAs are included in this study. As shown in Table 1, the PD group was significantly younger (~ 7 years) than the HOA group, but did not differ in education. Cognitive screening scores show the PD group scored 1–2 points lower than the HOA group, which was not significant for DRS scores, but represented a significant difference for MMSE. However, as seen in Table 1, the mean MMSE score for the PD group was greater than 29; thus, the actual difference was minimal. All participants were ambulatory without assistance and had normal or corrected to normal vision.

**Table 1:** Descriptive information for participants.

Measure	HOA (SD)	PD (SD)	p
N	20	28	
Age*	72.74 (9.33)	65.64 (10.08)	.011
Education (yrs.)	18.42 (2.01)	17.14 (4.07)	.212
DRS-2 (maximum = 145)	141.63 (1.77)	140.18 (3.50)	.102
MMSE	30.0 (0)	29.16 (1.2)	.001
Schwab & England* (max = 100)	--	88.50 (8.75)	
UPDRS	--	40.81 (13.14)	
Hoehn & Yahr	--	2.29 (0.34)	

*t(45) = 2.657, *p* = .011

Participants in the PD group had a diagnosis of idiopathic Parkinson’s disease, according to UK Brain Bank criteria, which was confirmed by a movement disorders neurologist. They had a modified Hoehn and Yahr scale score between 1 and 3 in the “on” medication state [21], and a stable response to anti-parkinsonian and/or psychotropic medication. Participants with signs of secondary or atypical Parkinsonism, or severe, unpredictable episodes of motor fluctuation were excluded from enrollment. In addition, potential participants with a history of falls (i.e. a score >1 in the fall item of the Unified Parkinson’s Disease Rating Scale Part II) were also excluded under advisement of the Institutional Review Board to minimize risk to participants.

Individuals from either group using medications known to interfere with cognitive functions, symptoms of mild cognitive impairment or dementia (MMSE score < 25), or a history of psychiatric disturbance (e.g. major depressive disorder or generalized anxiety) or cardiovascular disease were excluded from the study. This study was approved by the Health Sciences Institutional Review Board of the University of Florida (protocol #161–2010), and all participants completed an Informed Consent form approved by this body.

### Procedure

All participants with PD were evaluated using the Movement Disorders Society’s revision of Unified Parkinson’s Disease Rating Scale (UPDRS) [22] to determine disease severity. The complete UPDRS, including all subsections, was videotaped prior to testing for later evaluation by an independent clinician rater. All participants completed the Mattis Dementia Rating Scale Revised (DRS; [23]) and MMSE [24] to measure cognitive impairment.

### Cognitive tasks

Participants completed a battery of twelve cognitive tests (Table 2) in the on-medication state twice, while sitting in a quiet room and while cycling on a stationary bicycle. Order of single and dual task sessions was counterbalanced. The cognitive tests covered six cognitive domains: processing speed, controlled processing, visual processing, verbatim memory, working memory, and executive function [6,25,26]. Each task was assigned, a priori, a hypothesized difficulty level based on demands for attention and complex cognitive processing [25,27]. Tasks required only oral responses to ensure safety while cycling. Stimuli were self-paced and presented on a projection screen by a laptop computer using Direct-RT software [28]. Responses were recorded by Direct-RT. Each trial was scored later by trained research assistants for accuracy and response times using Audacity software [29]. Note that the primary dependent variable for each cognitive task is listed in Table 3. Stimulus lists were counterbalanced across subjects. Tasks were presented in the same order during single and dual task sessions, alternating difficult and easy tasks to minimize cognitive fatigue and ensure similar task order effects across conditions. Total cycling time ranged from 33 to 50 minutes, due to differences in response times across tasks, with no dual task lasting more than 5 minutes.

**Table 2:** This table lists the cognitive tasks by domain and hypothesized difficulty according to Colcomb and Kramer (2003) and Al-Yahya et al. (2011).

Domain	Hypothesized Difficulty	Task	Task Demands	Task Description	Primary Dependent Variable
Processing speed	1	Simple Visual Attention	Alerting	Say “Go” when you see the blue star, variable inter-stimulus interval. (N Trials = 20)	Response Time
	1	Articulation speed	Sustained attention, Articulation	Say “Pa” as many times as you can in 10 seconds. (10 seconds)	Number produced
Controlled Processing	2	0-back	Selective attention, sustained attention	Say “Yes” when the current figure matches a pre-specified target figure; otherwise say “No.” (Trials N = 40; 10 are “yes” trials)	Response Time for “Yes” trials
	2	Stroop Colors	Lexical access	Name the color of a set of Xs. (N Trials = 20)	Response Time
	2	Stroop Color Word	Inhibition, Lexical access	Name the color of the font a color word is shown in. (N Trials = 20)	Response Time
Visual processing	3	Digit Symbol Substitution	Visual search Visual comparison	Find the featured symbol in an array and say the number associated with it. (N Trials = 20)	Response Time
Verbatim Memory	4	1-back	Comparison, Updating	Say “Yes if the current figure matches the previous one; otherwise say “No.” (Trials N = 40; 10 are “yes” trials)	Response Time for “Yes” trials
	4	Digit span forward	Encoding, Recall	Repeat increasingly long lists of numbers. (N trials variable)	Number of lists recalled correctly
Working Memory	5	Visual memory span	Encoding, Updating, Recall, Comparison	Say “Yes” if an array contains the same set of 1–4 figures in the same order as just seen; otherwise say “No.” (N Trials = 16)	Percent Correct
	5	Digit span backward	Encoding, Rehearsal, Manipulation	Repeat increasingly long lists of numbers in reverse order of presentation. (N trials variable)	Number of lists recalled correctly
Executive Function	6	2-back	Encoding, Comparison, Set-Shifting, Updating	Say “Yes” if the current figure matches the figure presented 2 screens before. Otherwise say “No.” (Trials N = 40; 10 are “yes” trials)	Response Time for “Yes” trials
	6	Operation Span	Encoding Rehearsal, Dual tasking, Inhibition, Recall	Repeat and memorize 6 letters. Verify 0–4 simple math problems. Recall as many letters as you can in the correct order. (N Trials = 20)	Average number of letters recalled in sequence

In addition, hypothesized task demands (Lezak, Howieson, & Loring, 2004), number of trials, and dependent variables for each task are provided.

**Table 3:** Means and standard deviations for performance on cognitive tasks during single and dual task conditions.

		PD		HOA	
Task		Single task (SD)	Dual task (SD)	Single task (SD)	Dual task (SD)
Simple Attention	Response time	448 (152.1)	453 (131.84)	415 (58.0)	423 (65.7)
Articulation speed ^◊^	Number	53.0 (11.1)	53.6 (11.0)	57.6 (8.2)	58.6 (6.8)
0-back	Response time ^◊^	631 (128.9)	581 (89.4)	568 (87.2)	551 (95.7)
	Accuracy	98.8 (3.2)	99.5 (2.7)	99.4 (2.4)	1.0 (0.0)
Stroop Colors	Response time (ms)	605 (79.2)	655 (125.6)	618 (98.2)	614 (75.1)
	Accuracy ^Δ^	99.3 (2.4)	99.8 (0.9)	99.4 (1.6)	99.7 (1.1)
Stroop Color Word	Response time (ms)^◊^ ^Δ^	929 (205.3)	933 (254.5)	869 (112.7)	849 (97.0)
	Accuracy	93.5 (14.2)	93.0 (10.5)	94.1 (3.8)	93.0 (22.9
Digit Symbol	Response time (ms)^Δ^ ^§^	2699 (722.6)	2558 (576.6)	2521 (481.3)	2477 (431.8)
	Accuracy	90.5 (11.2)	89.8 (11.8)	96.0 (5.2)	94.8 (4.3)
1-back	Response time (ms)^Δ^	745 (247.5)	735 (178.2)	730 (150.9)	737 (364.8)
	Accuracy ^◊^	92.9 (11.7)	90.0 (16.9)	97.9 (5.4)	96.6 (7.5)
Digit span forward	Number of lists	8.5 (2.0)	8.1 (2.5)	8.8 (2.7)	9.1 (2.9)
Visual memory	Response time (ms)	2415 (956)	2228 (811)	2419 (1280)	1943 (638)
	Accuracy ^◊^ ^Δ^	69.2 (17.0)	72.9 (16.4)	79.1 (13.2)	76.3 (18.4)
Digit span backward	Number of lists	6.7 (1.9)	6.3 (1.8)	7.1 (2.4)	7.0 (2.2)
2-back	Response time (ms)^◊^	1168 (539)	1100 (780)	976 (329)	796 (185)
	Accuracy ^¥^ ^◊^	80.0 (23.9)	70.6 (23.2)	89.1 (13.5)	81.1 (7.5)
Operation Span ^◊^	Number of letters	3.7 (1.1)	3.6 (1.1)	4.5 (1.3)	4.7 (1.3)

Note that the primary dependent is underlined. All response times are in milliseconds. Accuracy is reported as percent correct, unless otherwise noted.

^◊^Groups differ p<.05

^§^ Significant dual task benefit

^¥^ Significant dual task cost

^Δ^ Significant age effect

### Cycling

Cycling was performed on the same stationary bicycle for all participants against minimal resistance. Seat height was adjusted for each participant. Reflective markers were attached to the ankle and heel of each foot. Cycling speed (rotations per minute, RPM) was collected using a 10-camera motion capture system (Vicon Motion Capture System, Los Angeles, CA). Immediately before the dual task session, single task (baseline) cycling speed was collected while participants cycled for two minutes at a self-selected comfortable rate in front of the projection screen showing the title of the study. Thus, single task, baseline cycling was always collected before dual task cycling. After participants heard the directions for each task, they were told to begin cycling, and stimulus presentation began immediately. No instructions were given regarding task prioritization. Participants rested between dual tasks and did not cycle during instructions. Heart rate was monitored throughout.

### Outcome Measures

The primary dependent variable for the initial ANOVA was mean cycling speed (RPMs). For subsequent analyses, the dependent variable was the Dual Task Effect (DTE) on cycling, that is, the percent change in dual task cycling speed relative to baseline cycling speed, calculated as 100 x ((dual task (RPMs)—baseline(RPMs))/baseline (RPMs) [3,30]. Negative values signified performance declined during the dual task (dual task costs); positive values signified that performance improved in the dual task (dual task benefits).

### Statistical Analysis

Initially, a 13 task (baseline + 12 cognitive tasks) by 2 group (PD, HOA) repeated measures MANOVA was used to assess the effects of the twelve cognitive tasks on cycling speed relative to baseline, single task cycling. Planned pair-wise comparisons using Bonferroni corrections compared performance between baseline cycling speed and cycling speed during the cognitive tasks. Two-way ANCOVAs (group by single/dual task) covarying age were used to assess dual task effects in cognitive tasks. All relevant cycling and cognitive data are available from the University of Florida Institutional Repository (http://ufdc.ufl.edu/ufirg), indexed as “Dual_Task_Benefits_on_Cycling_dataset”.

Finally, a hierarchical regression was chosen to examine the prediction regarding the relationship between group, task difficulty and DTEs, because it could determine the separate contributions of hypothesized task difficulty and group to the magnitude of DTEs that were found in the different dual tasks [31]. The dependent variable for the regression was the mean DTE on cycling during each dual task for each group. For the hierarchical regression, a dummy variable was constructed to identify the group means of the PD participants (coded as a 1) versus the group means of the HOA group (coded as 0). The independent variable, hypothesized task difficulty, was entered in the first step of the model, and group was entered in the second step. A significant effect of hypothesized task difficulty would signify that DTEs changed reliably with task difficulty. A significant group effect would mean that the intercepts for the two groups were different, that is, the overall magnitude of DTEs differed between groups.

Significance was set at α <0.05. All analyses were completed using IBM SPSS Statistics, version 22 (IBM, 2013).

## Results

### Cycling Performance

Group mean RPMs and DTEs on cycling speed during the cognitive tasks are presented in Table 4. Cycling speed in both groups increased when concurrently performing most cognitive tasks. The MANOVA comparing the cycling speeds of participants during the various cognitive tasks to baseline cycling speed between groups revealed a significant multivariate effect of group [*F*(1, 46) = 4.933, *p* = .031, η_p_^2^ = .10]. Overall, the HOA group cycled faster than people with PD. As hypothesized, mean cycling speed also differed significantly across cognitive domains [*F*(12, 35) = 13.608, *p* < .001, η_p_^2^ = .82]. Planned comparisons revealed that cycling speeds for six tasks were significantly faster than mean baseline speed: the simple attention task, articulation speed, 0-back, 1-back, Stroop colors, and Stroop color words. The interaction between group and task was not significant [*F*(12,552) = 1.469, *p* = .132, η_p_^2^ = .03]. The same analysis was subsequently recomputed as a MANCOVA covarying age, which can alter the magnitude of DTEs [3]. The effect of age was significant [*F*(1, 44) = 10.509, *p* = .002, η_p_^2^ = .19]; older age was associated with lower cycling speeds. The effect of group remained significant [*F*(1, 44) = 11.410, *p* = .002, η_p_^2^ = .21], after controlling for age.

**Table 4:** Cycling speeds and dual task costs (DTEs) on cycling performance.

	Cycling Speed (RPM)		Dual Task Effects (%)	
Task	PD (SD)	HOA (SD)	PD (SD)	HOA (SD)
Single task, baseline	48.6 (12.8)	53.9 (15.7)		
Simple Attention	55.7 (14.3)*	64.7 (17.4)*	+16.3 (18.8)	+25.6 (29.1)
Articulation speed	53.0 (12.5)*	64.7 (18.3)*	+12.8 (27.2)	+26.3 (42.5)
0-back	52.7 (12.5)*	64.2 (16.2)*	+11.9 (20.1)	+23.5 (30.0)
Stroop Colors	52.0 (13.4)*	60.4 (17.9)*	+ 9.5 (13.4)	+13.4 (16.8)
Stroop Color Word	52.2 (13,6)*	60.8 (18.1)*	+ 9.3 (19.6)	+15.7 (28.2)
Digit Symbol	48.8 (12.9)	60.2 (17.7)*	+ 3.5 (23.2)	+15.2 (28.2)
1-back	51.1 (12.2)*	59.5 (17.1)*	+ 7.8 (14.9)	+12.7 (18.9)
Digit span forward	47.3 (14.7)	57.8 (18.7)	- 0.4 (23.1)	+ 9.4 (29.3)
Visual memory span	48.3 (12.3)	55.0 (16.8)	+ 1.5 (11.6)	+ 2.6 (12.4)
Digit span backward	46.3 (14.7)	56.1 (18.2)	- 1.0 (26.0)	+ 6.1 (25.8)
2-back	49.6 (13.4)	60.1 (18.7)*	+ 3.8 (18.2)	+ 14.7 (32.1)
Operation Span	47.3 (14.4)	57.0 (18.3)	- 1.0 (0.9)	+ 7.8 (24.3)
Mean DTC			+ 6.2 (16.8)	+ 14.3 (23.4)

Positive values indicate faster cycling in the dual task than during single task, baseline cycling. Tasks are listed in the hypothesized order of difficulty.

* RPM was significantly greater than baseline after Bonferroni corrections.

### Cognitive Performance

Regarding cognitive performance (Table 3), participants performed significantly worse during the dual task in only one cognitive task, 2-back. There were significant positive DTEs (i.e., dual task benefits) on response times in the Digit Symbol and Zero-back tasks, but these were no longer significant when age was covaried. No other dual task effects on cognitive performance measures, primary or secondary, were significant. Thus, for the most part, performance on cognitive tasks during cycling mirrored single task cognitive performance. Furthermore, correlations were not significant between cognitive task order, cycling speed, and cognitive performance.

### Effects of Cognitive Task Difficulty

**Fig 1:**
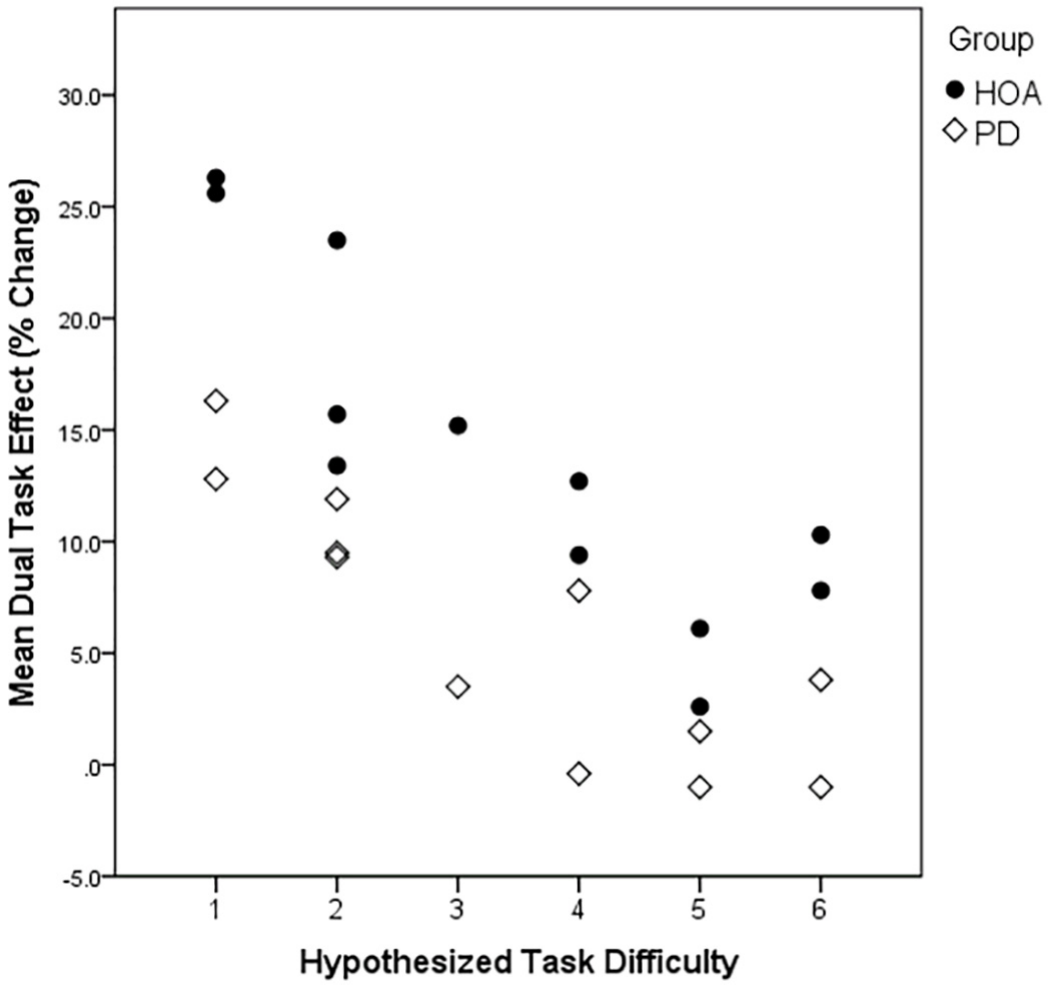
Scatterplot showing the mean Dual Task Effects (percent change) on cycling for each group plotted by the hypothesized difficulty of the secondary cognitive tasks (see Table 2). Positive values indicate faster cycling during dual tasks.

The hierarchical linear regression [31] using mean group DTEs per task as dependent variables found that hypothesized task difficulty (Table 2) and group significantly predicted performance. As illustrated in Fig 1, the hypothesized difficulty of the cognitive domain accounted for 53.6% of variance in mean DTEs on cycling across tasks [*r* = -.73, *p* < .001; B = -3.19, β = -.732]. Participant group accounted for an additional 26.5% of the variance [*F change* = 28.103; *p* < .001; B = -7.88, β = -.515], indicating that the intercept of the regression lines for the two groups were significantly different. Total variance accounted for by this model was 80.2%. Tasks of the greatest difficulty had the largest negative effect on DTEs, calculated from an intercept of +24.96%. Thus, actual DTEs were positive and greatest in the easiest tasks, and the magnitude of DTEs was lower for people with PD than HOAs.

## Discussion

Unexpectedly, cycling speed actually increased during dual tasks, leading to dual task *benefits* rather than the expected dual task *costs* for both populations. Further, DTEs of the PD group were less than those of the HOAs, rather than greater. Simultaneously, performance in cognitive tasks in the dual task condition did not differ from single task performance with three exceptions, performance in two cognitive tasks improved (although the difference was not significant when age was covaried) and performance in one cognitive task declined in both groups. These findings cannot be explained by current theories in which dual task effects are attributed to the cognitive demands of the two concurrent tasks exceeding available cognitive resources. We conclude that additional factors must be playing a role in dual task situations.

### Accounting for Dual Task Benefits

Research on acute exercise, which examines cognitive performance during dual tasks when exercise levels are held constant, offers a partial explanation. The acute exercise literature posits that exercise-related arousal increases the amount of processing resources available for the concomitant, “secondary” cognitive task, sometimes leading to improved cognitive performance during exercise relative to performance with no exercise task [32]. However, because the intensity of the exercise is always controlled in acute exercise research, their findings cannot address the improvements in cycling performance found in the current study.

To account for the findings of the current study and unify the findings of the acute exercise and the dual task research, these data suggest that a model of dual task performance should incorporate the arousing effects of the cognitive tasks on motor performance as well as physiological arousal due to the exercise itself [33,34]. Specifically, the perception that performing motor and cognitive tasks concurrently will be challenging could increase overall arousal [35]. Crucially, both cognitive and exercise-related arousal have been associated with increases in cognitive resources, improvements in speed, and greater efficiency of cognitive and motor responses [32,33,36]. Moreover, both physiological and cognitive arousal have been attributed to increased release of catecholamines, particularly epinephrine, norepinephrine and dopamine [33–36].

Specifically, we hypothesize that when faced with dual tasks, the perception that performing the tasks concurrently will be novel and challenging could increase cognitive arousal [36], triggering the release of dopamine and norepinephrine. This increased production of catecholamines would increase the availability of supplementary cognitive resources that, in turn, facilitates performance in both the cognitive and motor task. Consequently, dual task performance will be based on this increased level of cognitive resources, as modulated by the attentional demands of the concurrent tasks. Based on this reasoning, we posit an expansion of Kahneman’s account of dual task performance [9] and the exercise-related arousal model discussed in the acute exercise literature [10,34], which we call the Arousal and Attentional Demands (AAD) model of dual task performance. According to the AAD model, when the increase in attentional resources due to cognitive and physiological arousal matches the actual demands of the combined dual tasks, performance on both tasks can be maintained with no observable dual task cost. Dual task costs *only* appear when the additional arousal due to the dual tasks does not provide adequate cognitive resources to maintain performance levels in both tasks. Conversely, according to the AAD model, when the cognitive demands of the combined dual tasks are less than anticipated, the increased arousal can result in dual task benefits on performance rather than dual task costs, as found in the current study.

The catecholamine-dependent arousal hypothesized by the AAD predicts that people with low levels of dopamine, norepinephrine and epinephrine will show significant differences in dual task effects from control subjects. PD leads to deterioration of dopaminergic input to frontal and subcortical regions [37,38] which would necessarily affect levels of norepinephrine and epinephrine [39]. Therefore, people with PD might be expected to demonstrate limited catecholamine-dependent arousal when challenged with a dual task. This reasoning is consistent with the findings of the current study. The PD group exhibited DTEs below those of HOAs in all 12 tasks in the current study (mean difference 8.1%, range 3.9%–13.5%). In studies with more difficult motor tasks such as walking or maintaining balance, the AAD model would predict that participants with PD would benefit less from dopamine-dependent arousal and, consequently, evince greater dual task impairments than HOAs, which is indeed the typical finding in the literature [13].

Interestingly, the mechanism that we hypothesize is responsible for our findings, the release of dopamine and/or norepinephrine due to challenging, exogenous stimuli, is a similar mechanism to that postulated to underlie kinesia paradoxica in PD [40,41]. Kinesia paradoxica is the phenomenon in which motor performance is facilitated by a threatening event, such as moving to avoid an approaching object. Kinesia paradoxica effects are believed to be due to arousal mediated primarily by stress, leading to increased production of noradrenaline and epinephrine [34]. Alternatively, contextual or psychological factors may also trigger a release of striatal or mesolimbic dopamine that facilitates motor performance [40,42]. Importantly, these accounts are not mutually exclusive; both may play a role in the phenomenon [35]. It is possible that kinesia paradoxica and dual task performance may represent different instances of similar arousal-related, catecholamine-dependent, physiological phenomena that differ primarily in magnitude.

While we have adopted Kahneman’s approach to dual task performance, in which processing resources are shared between the ongoing motor and cognitive tasks [9, 10], as the foundation for the AAD, other explanations for dual task effects have been offered. In particular, it has been suggested dual task performance involves switching attention between tasks [43], so dual task costs actually represent switch costs in this explanation. To account for the current findings, this conceptualization of dual task performance would also need to invoke the effects of cognitive and exercise-related arousal; however, it would have more difficulty accounting for the changes in cycling performance with increasing task difficulty. Thus, accounting for the findings of the current study, as well as findings from the acute exercise and dual literatures, would be a significant challenge for the task-switching account of dual task performance.

### Contributions of Task Difficulty to Dual Task Performance

Why should cycling, in which we found dual task benefits, differ so decisively from walking and balance, in which dual task costs are endemic? Cycling on a stationary bicycle differs fundamentally from walking. Walking is a weight-bearing exercise that requires weight shifting both laterally and sagittally [44], leading to a much higher demand for dynamic postural control than stationary cycling [45]. Stationary cycling does not require continuous monitoring of movement in both legs, because the motions of the legs are linked together [19] thus providing continuous cuing for the opposite leg via kinesthetic feedback as the contralateral pedal rises [18]. Thus, it is possible that the effects reported here may be specific to cycling and absent in tasks like walking that rely more on cognitive input. However, the relative difficulty of walking and cycling remains controversial. For example, Yogev-Seligman and colleagues [45] report that HOAs and people with PD experience greater dual task costs during cycling compared with walking in some but not all gait measures. On the other hand, Lambourne and Tomporowski [32] conclude that dual task benefits on cognitive tasks in acute exercise are more likely while cycling, while dual task costs on cognitive tasks are more prevalent during treadmill walking, findings that are consistent with the AAD model.

Consistent with our predictions, DTEs in this study were mediated by the difficulty of the cognitive task: The more complex the cognitive task, the less facilitation there was of cycling speed. Based on the AAD model, these graded effects of cognitive tasks can be interpreted as representing a reduction in cycling speed from the new dual task baseline, reset by arousal resulting from the dual tasks. From this perspective, compared to the easiest cognitive tasks which showed significant dual task benefits, the most difficult tasks resulted in approximately a 22% decrease in cycling speed for HOAs and a 14% decrease in cycling speed for the PD group. The pervasiveness of dual task benefits during the easiest tasks suggests that pairing cycling with simple, fast-paced tasks may be an effective way to increase the intensity of exercise in people with age-related or pathological slowing.

A potential limitation of this study is that participants with PD were tested only on medication. Future studies that include evaluation of PD performance in the off-medication state will provide further information regarding the relationship between PD, cycling and cognitive performance, while providing a good test for the AAD model of dual task performance. Another limitation is that participants with PD were mildly to moderately affected by the disease (mean H&Y score = 2.3), and all participants were cognitively intact. It remains unknown whether similar dual task benefits would be experienced by newly diagnosed or more severely impaired people with PD or by people with cognitive impairment.

## Conclusions

This study unexpectedly demonstrated that the cycling speed of HOAs and people with PD increased when paired with easy cognitive tasks in a dual task paradigm. Further, it demonstrated that increases in cycling speed were significantly predicted by cognitive task difficulty. Finally, it found reduced dual task benefits on cycling speed in PD relative to those in HOAs.

While other accounts of this phenomenon are certainly possible, the AAD model of dual task effects provides a testable hypothesis to account for the results of this study which allows us to reconcile our findings with the dual task and acute exercise research. This model leads to testable predictions for research in both fields especially with respect to the effects of cognitive tasks of differing difficulty on motor performance and the magnitude of dual task effects in people with PD.

The AAD model includes a catecholamine-dependent arousal component that enhances performance in response to challenging cognitive or physical circumstances. According to this model, the combination of relatively low motor demands during cycling and low cognitive demands during simple, fast-paced cognitive tasks resulted in dual task benefits on cycling performance in both healthy adults and those with PD. Importantly, dual task benefits were significantly lower in people with PD than in HOAs, consistent with the proposed AAD model. We suspect that dual task challenges with relatively easy motor and cognitive tasks may have therapeutic relevance for both age-related and pathological slowing.
